# DNA-Dependent RNA Polymerase Detects Hidden Giant Viruses in Published Databanks

**DOI:** 10.1093/gbe/evu128

**Published:** 2014-06-13

**Authors:** Vikas Sharma, Philippe Colson, Roch Giorgi, Pierre Pontarotti, Didier Raoult

**Affiliations:** ^1^Aix-Marseille Univ., Unité de Recherche sur les Maladies Infectieuses et Tropicales Emergentes (URMITE) UM63 CNRS 7278 IRD 198 INSERM U1095, Marseille, France; ^2^Aix-Marseille Univ., I2M UMR-CNRS 7373, Evolution Biologique et Modélisation, Marseille, France; ^3^Fondation Institut Hospitalo-Universitaire (IHU) Méditerranée Infection, Pôle des Maladies Infectieuses et Tropicales Clinique et Biologique, Fédération de Bactériologie-Hygiène-Virologie, Centre Hospitalo-Universitaire Timone, Assistance Publique-Hôpitaux de Marseille, Marseille, France; ^4^Aix-Marseille Université, UMR S 912 (SESSTIM), INSERM, IRD, Marseille, France; ^5^Assistance Publique–Hôpitaux de Marseille, hôpital Timone, Service Biostatistique et Technologies de l’Information et de la Communication, Marseille, France

**Keywords:** DNA-dependent RNA polymerase, giant virus, “Megavirales”, Mimivirus, dark matter, metagenomes, domains of life, environment

## Abstract

Environmental metagenomic studies show that there is a “dark matter,” composed of sequences not linked to any known organism, as determined mainly using ribosomal DNA (rDNA) sequences, which therefore ignore giant viruses. DNA-dependent RNA polymerase (RNAP) genes are universal in microbes and conserved in giant viruses and may replace rDNA for identifying microbes. We found while reconstructing RNAP subunit 2 (RNAP2) phylogeny that a giant virus sequenced together with the genome of a large eukaryote, *Hydra magnipapillata*, has been overlooked. To explore the dark matter, we used viral RNAP2 and reconstructed putative ancestral RNAP2, which were significantly superior in detecting distant clades than current sequences, and we revealed two additional unknown mimiviruses, misclassified as an euryarchaeote and an oomycete plant pathogen, and detected unknown putative viral clades. We suggest using RNAP systematically to decipher the black matter and identify giant viruses.

## Introduction

Current knowledge on microbiology is evolving rapidly as metagenomics, single-cell genomics, and culturomics advance (Edwards and Rohwer 2005; Lagier et al. 2012; Rinke et al. 2013). Nonetheless, these technologies leave unclassified a dark matter that comprises as much as 70% of sequences obtained by metagenomics and at least 16% of microbes observed by electron microscopy in the gut (Edwards and Rohwer 2005; Suttle 2005; Lagier et al. 2012; Hugon et al. 2013). Culture and single-genome sequencing allow for the recovery of the genomes of unidentified microbes, then reannotating metagenomic databases (Lagier et al. 2012; Rinke et al. 2013). However, in most metagenomic studies, identifications of microbes have been based on 16S ribosomal DNA (rDNA) similarity (Eckburg et al. 2005; Reyes et al. 2010). This approach results in poor identification of atypical bacterial phyla and in neglecting giant viruses that are also microbes (Colson et al. 2012; Raoult 2013). Indeed, Mimivirus was long considered an intracellular bacterium, resisting for years identification by rDNA amplification (Raoult 2013), whereas pandoraviruses were identified morphologically 15 years ago as putative intracellular eukaryotic symbionts before being classified as viruses (Philippe et al. 2013). In addition, as they lack ribosomal genes, these giant viruses are not part of the rDNA tree that encompasses the three currently defined domains of life, namely Archaea, Bacteria*,* and Eukarya.

**Fig. 1.—:**
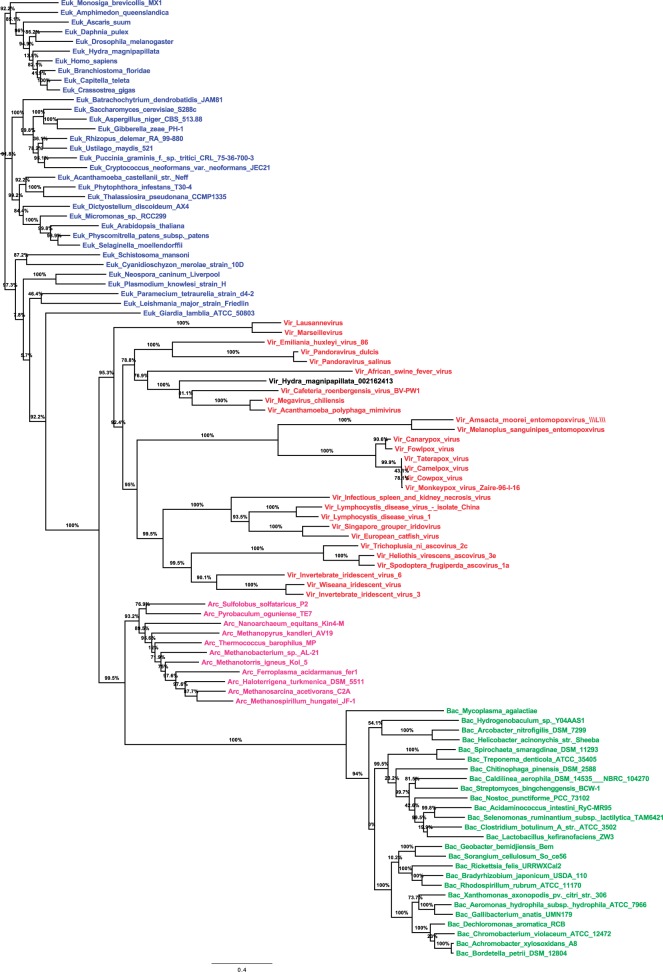
Phylogeny reconstruction using the maximum likelihood method for DNA-dependent RNAP2. The tree was based on 99 sequences and 420 positions. Members of Megavirales, Bacteria, Archaea, and Eukarya are indicated in red, green, pink, and blue, respectively; the extra *Hydra magnipapillata* subunit is indicated in black. Scale bar represents the number of estimated changes per position. See also supplementary figure S1, Supplementary Material online.

**Fig. 2.—:**
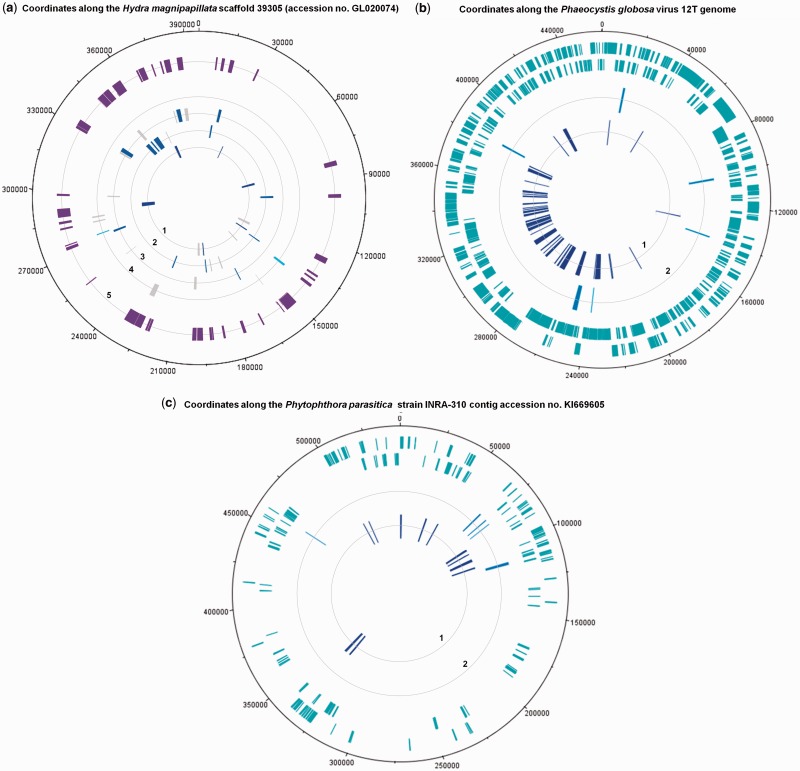
Circular representations showing best and significant BLASTp hits for protein-coding predicted genes detected using GeneMarkS (Besemer and Borodovsky 2005) from a scaffold obtained from *Hydra magnipapillata* (GenBank accession number GL020074.1) (*a*) and for protein sequences downloaded from GenBank for the Marine Group II euryarchaeote SCGC AB-629-J06 (taxonomy ID: 1131268) draft genome (AQVM00000000) (*b*) and the contig accession no. KI669605 of *Phytophthora parasitica* strain INRA-310 (*c*). (*a*) Best hits corresponding to mimiviruses of lineages A, B, and C and distant mimiviruses (rings 1, 2, 3, and 4, respectively; blue) (Colson et al. 2012), and significant hits against a mimivirus at any rank among the 20 best hits (ring 5; purple) are mapped on the *Hydra magnipapillata* scaffold GL020074. Best hits corresponding to mimiviruses with a lower sequence coverage are colored in gray. (*b*) Best hits (ring 1) and other significant hits (ring 2) for predicted proteins from the Marine Group II euryarchaeote SCGC AB-629-J06 draft genome against the *Phaeocystis globosa* virus 12T gene repertoire (chosen because it provided the highest number of hits among the mimiviruses) are mapped on this viral genome. Outer rings indicate *P. globosa* virus 12T ORFs in sens (outer) and antisense (inner) orientations. (*c*) Best (ring 1) and other significant hits (ring 2) corresponding to a Megavirales member are mapped on the contig accession no. KI669605 of *Phy. parasitica* strain INRA-310. Outer rings indicate *Phy. parasitica* ORFs in sens (outer) and antisense (inner) orientations.

A relevant alternative to rDNA for microbe identification is DNA-dependent RNA polymerase (RNAP) genes. RNAP is a good alternative to 16S rDNA for bacterial identification and was described to provide similar or greater phylogenetic resolution (Case et al. 2007; Adekambi et al. 2009). They are more refractory to lateral gene transfers and usually present in a single copy in genomes, which avoids recombination and issues related to divergence between copies (Case et al. 2007; Adekambi et al. 2009). Moreover, RNAP is found in giant viruses (Boyer et al. 2010). We previously found that phylogeny of RNAP subunit 2 (RNAP2) and a few other informational genes delineated four branches that encompass members of Archaea, Bacteria, Eukarya, and large and giant DNA viruses that compose a monophyletic group named the nucleocytoplasmic large DNA viruses and were proposed to be reclassified in a new order, the “Megavirales” (Yutin et al. 2009; Boyer et al. 2010; Colson et al. 2013). In this work, we extend our previous phylogenetic study and clearly establish that RNAP delineates four branches of known organisms, that is, Archaea, Bacteria, Eukarya*,* and Megavirales, which were recently called “TRUCs,” an acronym for things resisting uncompleted classification, to emphasize that the three domain paradigm is not a comprehensive view of life (Raoult 2013). Eukaryotes encode three paralogous genes for each RNAP subunits 1 and 2, named RNAP I, II, and III, which have orthologs in bacteria, archaea, and Megavirales members (Werner and Grohmann 2011). Here, we used RNAP III because we found it was the most conserved, and we aimed to obtain an informative tree that included, in addition to RNAP2 homologs from megaviruses, those from a comprehensive, representative, and unbiased set of members from Bacteria, Archaea*,* and Eukarya. RNAP2 phylogeny reconstructions clearly delineated four branches (fig. 1 and supplementary fig. S1, Supplementary Material online). Interestingly, we detected that a sequence recovered from the draft genome of *Hydra magnipapillata*, a multicellular freshwater predatory cnidarian, was clustered with RNAP2 from amoeba-associated mimiviruses. The *H. magnipapillata* draft genome (≈1.0 gigabase pair large) had been obtained by a whole-genome shotgun procedure then 454 sequencing from laboratory strain 105 that was recloned from a single polyp and was described in 2010 in *Nature* (Chapman et al. 2010). Further analyses of the scaffold harboring the RNAP2-encoding sequence (scaffold 39305 [GenBank accession no. GL020074.1]) found that 21 of the 186 predicted genes had a mimivirus gene as best match, including one encoding a major capsid protein (fig. 2*a*; supplementary fig. S2 and table S1, Supplementary Material online), suggesting that a hidden Mimivirus relative was sequenced concurrently with the *Hydra* genome and unrecognized. This serendipitous finding indicated that RNAP2 is a promising tool to detect hidden giant viruses and prompted us to search for other similar misidentifications in the National Center for Biotechnology Information (NCBI) GenBank protein sequence database.

BLASTp analysis using RNAP2 from Megavirales allowed us to recover two additional RNAP2 that were clustered with giant viruses despite being not annotated as viral. The first sequence was from a marine group II Euryarcheota (strain SCGC AB-629-J06; accession no. NZ_AQVM00000000) and was among whole-genome shotgun sequences obtained from Lake Washington (WA, unpublished). The Euryarcheota is a phylum of mainly marine archaea, which are among the most abundant archaea in the oceans (Massana et al. 2000). BLASTp analysis for the 368 predicted proteins available in GenBank for this organism identified 92 genes having as best hit a mimivirus, mostly members of a distant group within the family Mimiviridae that encompasses *Phaeocystis globosa* virus and organic lake phycodnaviruses (Yutin et al. 2013) (fig. 2*b*; supplementary fig. S3 and table S2, Supplementary Material online). In contrast, no 16S rDNA sequence (nor capsid-encoding sequence) was found. The second sequence was from an eukaryotic plant pathogen, *Phytophthora parasitica*. Further analyses showed that a contig (accession no. KI669605; unpublished) from *Phy. parasitica* strain INRA-310 harbored both genes encoding RNAP subunits 1 and 2, and that 17 of its 120 predicted genes matched with a Megavirales member, as best match in 12 cases (fig. 2*c* and supplementary table S3, Supplementary Material online). Seven sequences were most related to African swine fever virus, including one homologous to its capsid gene. In the RNAP2 phylogenetic reconstruction, the marine group II euryarchaeote was clustered with *P. globosa* virus, whereas *Phy. parasitica* was clustered with African swine fever virus as a new distant member of the family Asfarviridae. Overall, the detection of these three overlooked giant viruses is a proof of concept that we can detect unknown viruses through comparative analyses of RNAP2 including in the genome of eukaryotes where they were not previously identified.

**Fig. 3.—:**
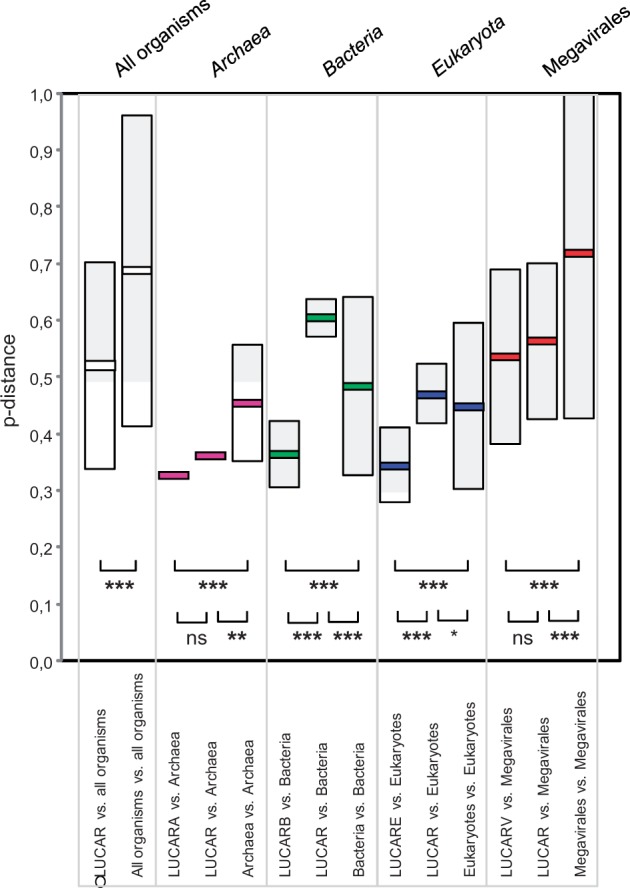
Plot of *p* distances obtained by pairwise comparisons between ancestral sequences constructed from DNA-dependent RNAP2 from members of Archaea, Bacteria, Eukarya, and Megavirales and these RNAP sequences, and between these RNAP sequences. LUCARA, LUCARB, LUCARE and LUCARV are the putative ancestral sequences of the RNAP2 from all members of the Archaea, Bacteria, Eukarya, and Megavirales, respectively; LUCAR is the putative ancestral sequence of these four reconstructed sequences. Boxes delimit ranges of *p* distances corresponding to the mean value ± two standard deviations within or across branches of life. Thick lines indicate the mean values. ***P value < 1e-6; **1e-6 < *P* < 1e-3; *P < 0.05; ns, not statistically significant.

Following our hypothesis of a fourth domain of life (Boyer et al. 2010), Wu et al. (2011) used RNAP sequences to “fish” into environmental sequence databases, mostly that from the Sorcerer II Global Ocean Sampling expedition, aiming to recover unknown organisms and identify new clades. These authors identified environmental sequences that were among the deepest branches within a domain of life or even between the branches encompassing Bacteria, Archaea, and Eukarya members and large viruses (only poxviruses being analyzed), and they assumed that these sequences could come from unknown viruses. We speculated here that obtaining reconstructed putative ancestral sequences of RNAP2 will increase the sensitivity to detect similarities to distant and unknown viruses. Thus, the distance may be theoretically greater between two distant members from a single phylum than between these sequences and the ancestor of this phylum. Therefore, we used MEGA5 software (Tamura et al. 2011) to construct the putative ancestral sequences (we named “mamas”) of the RNAP2 from all members of the Megavirales, Archaea, Bacteria, and Eukarya, and then LUCAR2, the putative ancestral sequence of these four reconstructed sequences. Such reconstruction of ancestral sequences accounts for the most probabilistic protein sequence for each phylogenetic node and has proved helpful to isolate new variants of sulfotransferases and paraoxonases (Alcolombri et al. 2011) and effective to provide a candidate Precambrian beta-lactamase sequence with catalytic efficiencies (Risso et al. 2013). Then, we tested our hypothesis by comparing the *p* distances measured between LUCAR2, the reconstructed ancestors for RNAP2 from given branches, and these RNAP2 to the *p* distances measured between the RNAP2. We found that mean *p* distances were majoritary significantly lower between reconstructed ancestors and RNAP2 than between these RNAP2 (fig. 3; supplementary fig. S4, Supplementary Material online). The distribution of p distance percentiles showed the same trend, with lower values for the 10th percentile, between reconstructed ancestors and RNAP2 as for between these RNAP2 (supplementary fig. S5, Supplementary Material online). Therefore, using RNAP2 mamas allows us to decrease the distance to the bottom level from each branch.

**Fig. 4.—:**
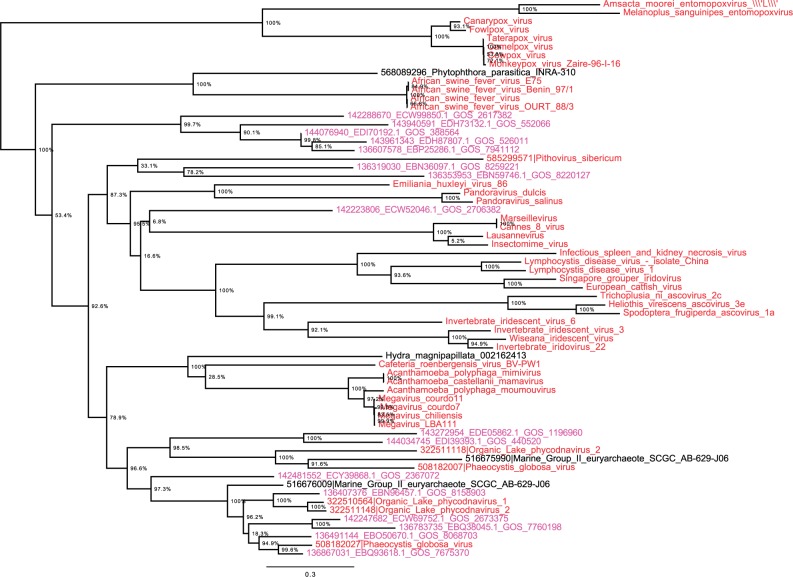
Phylogeny reconstruction using the maximum likelihood method for DNA-dependent RNAP2 from members of the Megavirales and metagenomic sequences fished by the reconstructed putative ancestral RNAP2 sequence. Members of Megavirales are indicated in red, whereas metagenomic sequences are indicated in pink and sequences related to giant viruses and previously misclassified are indicated in black. Scale bar represents the number of estimated changes per position.

Our next step was to use LUCAR2 as fishhook to recover distant homologs in the NCBI environmental database (env_nr) through BLASTp searches. This approach was fruitful for finding metagenome sequences composing new clades in the viral branch (fig. 4). Most of these metagenomic sequences were clustered within the Mimiviridae in the RNAP2 tree. Nodes within phylogeny were well supported with high confidence values. The novelty of these sequences was confirmed by phylogenetic analyses with other eukaryotic RNAP2 sequences and the best 100 BLAST hits, in addition to the same data sets. Indeed, these analyses showed that these metagenomic sequences were still clustered with viruses (supplementary fig. S6, Supplementary Material online). Noteworthy, we calculated that using RNAP2 would have found sequences from pandoraviruses (Philippe et al. 2013) as the 190th hit (e value, 7e-37; 33% identity along 352 amino acids). We did not recover the seven metagenomic sequences reported by Wu et al. (2011) as representing novel branches, possibly because we used more stringent parameters, including 70% query length coverage and an *e*-value cutoff of 1e-10. However, those sequences were among the BLAST results, even if positioned very far (3,306th rank, *e* value of 5e-10 and nucleotide identity of 26% for the best hit) (supplementary table S5, Supplementary Material online). These findings indicate that our analyses did not allow us to recover all the biodiversity present and prompt us to perform deeper analyses using different parameters. Also, we incorporated sequences we fished from environmental metagenomes into the RNAP2 sequence alignment previously reported by Wu et al. (2011) and found that the sequences we recovered formed a clade that branched deeply with the clade previously identified by Wu et al. as possibly composed of uncharacterized viruses, and two other new clades related to the archaeal branch that were not identified by Wu et al. (supplementary fig. S7, Supplementary Material online). Overall, these findings are proof that LUCAR2 is a powerful tool to recover sequences from unknown or unrecognized viruses and new viral clades.

The use of rRNA sequences for identification and classification of organisms has been extremely useful for 40 years (Case et al. 2007; Rinke et al. 2013). However, rendering 16S rDNA mandatory for the identification of organisms made us blind to microbes devoid of ribosomes, particularly giant viruses (Boyer et al. 2010; Raoult 2013; Colson et al. 2013). We demonstrated here that RNAP2 can be used to fish into sequence databases and identify organisms, in the same way that 16S rDNA has been used before. The use of RNAP2 allowed a more comprehensive recovery of living organisms that include giant viruses. As a proof of this concept, we identified giant viruses that were missed in sequence data sets, including among sequences published in *Nature* in 2010 (Chapman et al. 2010). Moreover, we used here for the first time reconstructed putative ancestral sequences to fish distant homologs in environmental metagenomic databases. Based on our results, the presence of organisms can be confirmed by polymerase chain reaction or fluorescence in situ hybridization in samples from which new sequences have been identified, to confirm the presence of currently unknown organisms. Overall, we introduced two concepts to decipher the “dark matter” in microbiota and provide a more comprehensive overview and classification of the biological diversity than rRNA alone. The first concept is to use RNAP2 as an “universal” probe to uncover new viral sequences, and the second one is to use putative ancestral sequences recreated for conserved genes to identify distant, undescribed viral clades. Thus, we need to sequence genomes from new organisms and develop concurrently new tools that are independent of rDNA to fish for unrecognized and unknown microbes in the sequence databases.

## Materials and Methods

### Collection of RNAP2 Homologous Sequences from the Three Cellular Branches of Life and from Members of the Proposed Order Megavirales

BLASTp searches for DNA-dependent RNAP2 homologous sequences from cellular organisms were performed using *Acanthamoeba polyphaga* mimivirus RNAP2 (gi: 311977620) as query sequence against the NCBI GenBank nonredundant protein sequence database (nr) with a number of target sequences limited to 20,000. Orthologous gene sequences to RNAP2 were obtained by Orthomcl (Li et al. 2003) using complete protein sets from the seven families of the proposed order Megavirales (Asfarviridae, Ascoviridae, Iridoviridae, Phycodnaviridae, Poxviridae, Marseilleviridae*,* and Mimiviridae) directly downloaded from the NCBI website (ftp://ftp.ncbi.nih.gov/genomes/Viruses/); RNAP2 from pandoraviruses, *P. globosa* viruses, and organic lake phycodnaviruses were collected from the NCBI GenBank nonredundant protein sequence database using BLASTp searches.

### Criteria for Selection of Sequences of RNAP 2 from Cellular Organisms

RNAP2 is highly conserved and may possess bulk of homologs in protein sequence databases. We aimed at obtaining an informative tree based on RNAP2 sequences from megaviruses and a comprehensive, representative, and unbiased set of species from Bacteria, Archaea*,* and Eukarya. Therefore, we selected RNAP2 sequences from members of Bacteria, Archaea*,* and Eukarya by using TimeTree, which is a professional resource where divergence time between species is reported on the molecular clocks based on studies published in peer-reviewed journals (Hedges et al. 2006). Precisely, species that were selected for this study were those that diverged around 500 Ma, a time point that allowed obtaining a reasonably comprehensive and representative set of members from Bacteria, Archaea*,* and Eukarya. The genomes of the majority of the organisms considered here are available and have been annotated. Taxon filter is a Java-based program that we used to filter out taxons and gi identifications from the BLAST results in XML format. Then, protein sequences from the selected species were downloaded directly from the NCBI GenBank nr database using the gi identifications. Finally, identical and partial sequences were removed manually after analyzing neighbor joining phylogeny and best BLAST hits.

### Phylogeny Reconstructions

Protein sequences were aligned using the Muscle program (Edgar 2004). Multiple sequence alignment trimming was done for the analysis of metagenomic sequences using the TrimAL program with the gappyout command, which calculates the gap percentage for the whole sequence alignment (Capella-Gutierrez et al. 2009). Phylogenetic reconstructions were performed using the maximum likelihood method with the Whelan and Goldman (WAG) substitution model. Confidence values were calculated by the Shimodaira–Hasegawa test using FastTree (Price et al. 2010). Phylogenetic trees were visualized by FigTree (http://tree.bio.ed.ac.uk/software/figtree/, last accessed May 1, 2014).

### Ancestral Sequence Reconstructions

Ancestral sequences were reconstructed for sequences from Archaea, Bacteria, Eukarya*,* and the proposed order Megavirales using the maximum likelihood method including the WAG substitution model and conducted by the MEGA5 software (Tamura et al. 2011).

### Comparative Analyses of *p* Distances between Reconstructed Ancestral Sequences for RNAP2 from Archaea, Bacteria, Eukarya, and Proposed Order Megavirales and These RNAP Sequences, and between These RNAP Sequences

*p* distances, that is, the proportions of amino acid sites at which two sequences to be compared are different, were obtained using the MEGA5 software (Tamura et al. 2011). Comparisons of mean *p* distances were performed using the OpenEpi software (www.openepi.com, last accessed May 1, 2014) with the analysis of variance test or the nonparametric Mann–Whitney *U* test when appropriate. Pairwise multiple comparisons (maximum number = 3) were performed between RNAP subunits 1 and 2 from members of Archaea, Bacteria, Eukarya*,* and Megavirales, using a Bonferroni correction to account for multiple testing. For an *α*-level of 0.05, applying this correction reduced the *P*-value threshold to 0.016 (two-sided test). In addition, for comparison of *p* distances, multidimensional scaling analysis was performed from matrices of Euclidian *p* distances, standardized, using the R software version 2.14.0 (R Development Core Team 2011).

### RNAP Homologous Sequence Detection from Metagenomic Databases

Sequences homologous to RNAP2 were searched for in environmental metagenomes by BLASTp against the NCBI GenBank environmental protein sequence database (env_nr) using LUCAR2 as query sequence, 70% query length coverage and an e-value cutoff of 1e-10 as stringent parameters and considering only RNAP homologs larger than 400 amino acids.

### Circular Representations of BLAST Matches between Protein Sequences of *H. magnipapillata*, Marine Group II Euryarchaeote SCGC AB-629-J06 and *Phy. parasitica*, and Mimiviruses and Other Megavirales Members

Putative protein sequences from scaffold 39305 (GenBank accession no. GL020074.1) of the draft genome of *H. magnipapillata* were predicted using the GeneMarkS software (Besemer and Borodovsky 2005); 337 ORFs were predicted including 186 equal to or larger than 100 amino acids in size. Protein sequences annotated from the draft genomes of marine group II euryarchaeote SCGC AB-629-J06 and *Phy. parasitica* were downloaded from the NCBI GenBank protein sequence database. The gene repertoires from all available Megavirales members were compiled into an in house database. BLASTp searches were run using protein sequences for each of the three organisms against the NCBI GenBank nonredundant protein sequence database to identify those having viral sequences as best hits (Altschul et al. 1990). Representations were created using DNAPlotter (http://www.sanger.ac.uk/resources/software/dnaplotter/, last accessed May 3, 2014).

## Supplementary Material

Supplementary tables S1–S5 and figures S1–S7 are available at *Genome Biology and Evolution* online (http://www.gbe.oxfordjournals.org/).

Supplementary Data
