# Supplementary material for: DNA-Dependent RNA Polymerase Detects Hidden Giant Viruses in Published Databanks
Source: Genome Biol Evol. 2014 Jun 13;6(7):1603–10. doi: 10.1093/gbe/evu128 (PMC4122926; doi:10.1093/gbe/evu128)
Supplement: Supplementary Data [file supp_6_7_1603__index.html]

DNA-dependent RNA polymerase detects hidden giant viruses in published databanks — DNA-Dependent RNA Polymerase Detects Hidden Giant Viruses in Published Databanks — Supplementary Data 

# DNA-Dependent RNA Polymerase Detects Hidden Giant Viruses in Published Databanks

## Supplementary Data

files

**Files in this Data Supplement:**

- Supplementary Data - pdf file
- Supplementary Data - pdf file
- Supplementary Data - pdf file
- Supplementary Data - tif file
- Supplementary Data - tif file
- Supplementary Data - tif file
- Supplementary Data - tif file
- Supplementary Data - tif file
- Supplementary Data - tif file
- Supplementary Data - tif file
- Supplementary Data - docx file
- Supplementary Data - xlsx file
- Supplementary Data - xlsx file
- Supplementary Data - xlsx file
- Supplementary Data - txt file
- Supplementary Data - xlsx file
