# Supplementary figures and images for: DNA-Dependent RNA Polymerase Detects Hidden Giant Viruses in Published Databanks
Source: Genome Biol Evol. 2014 Jun 13;6(7):1603–10. doi: 10.1093/gbe/evu128 (PMC4122926; doi:10.1093/gbe/evu128)

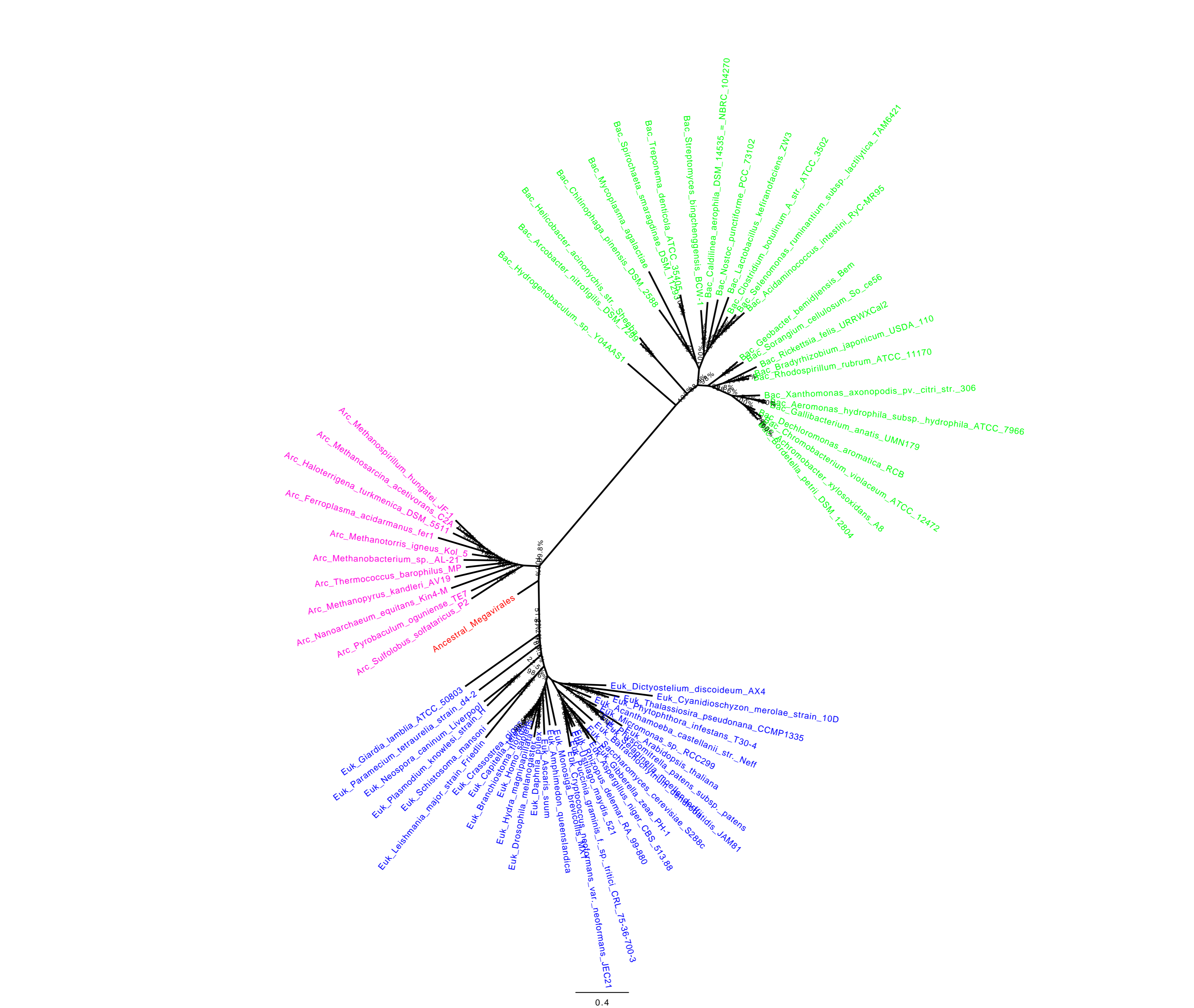

Supplement: Supplementary Data [file supp_evu128_RespRev_Sharma_Raoult_GBE_May2014_Rev_Tree_fig_1_plus_ancestral_1.pdf]

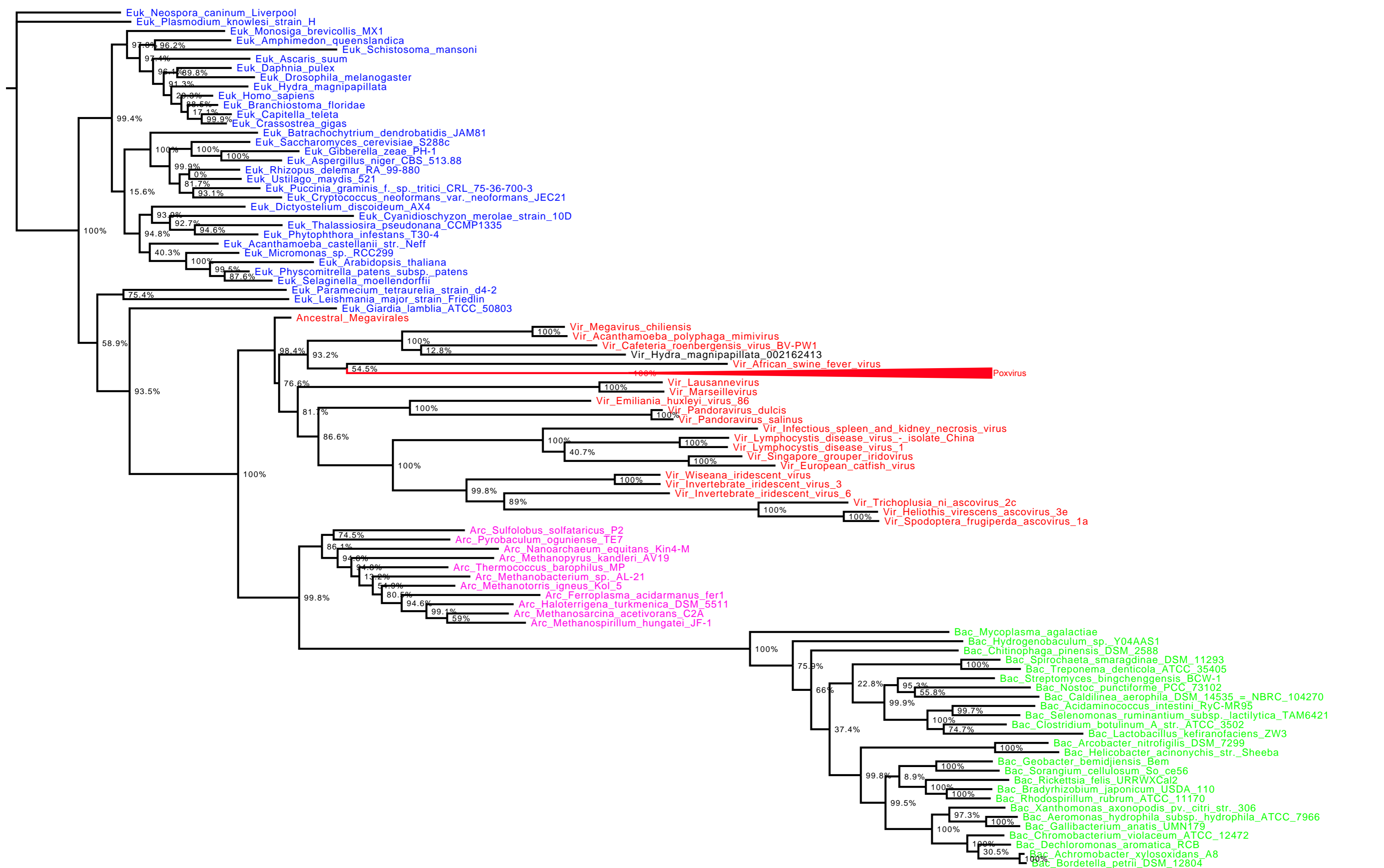

0.4

Supplement: Supplementary Data [file supp_evu128_RespRev_Sharma_Raoult_GBE_May2014_Rev_Tree_fig_1_plus_ancestral_2.pdf]

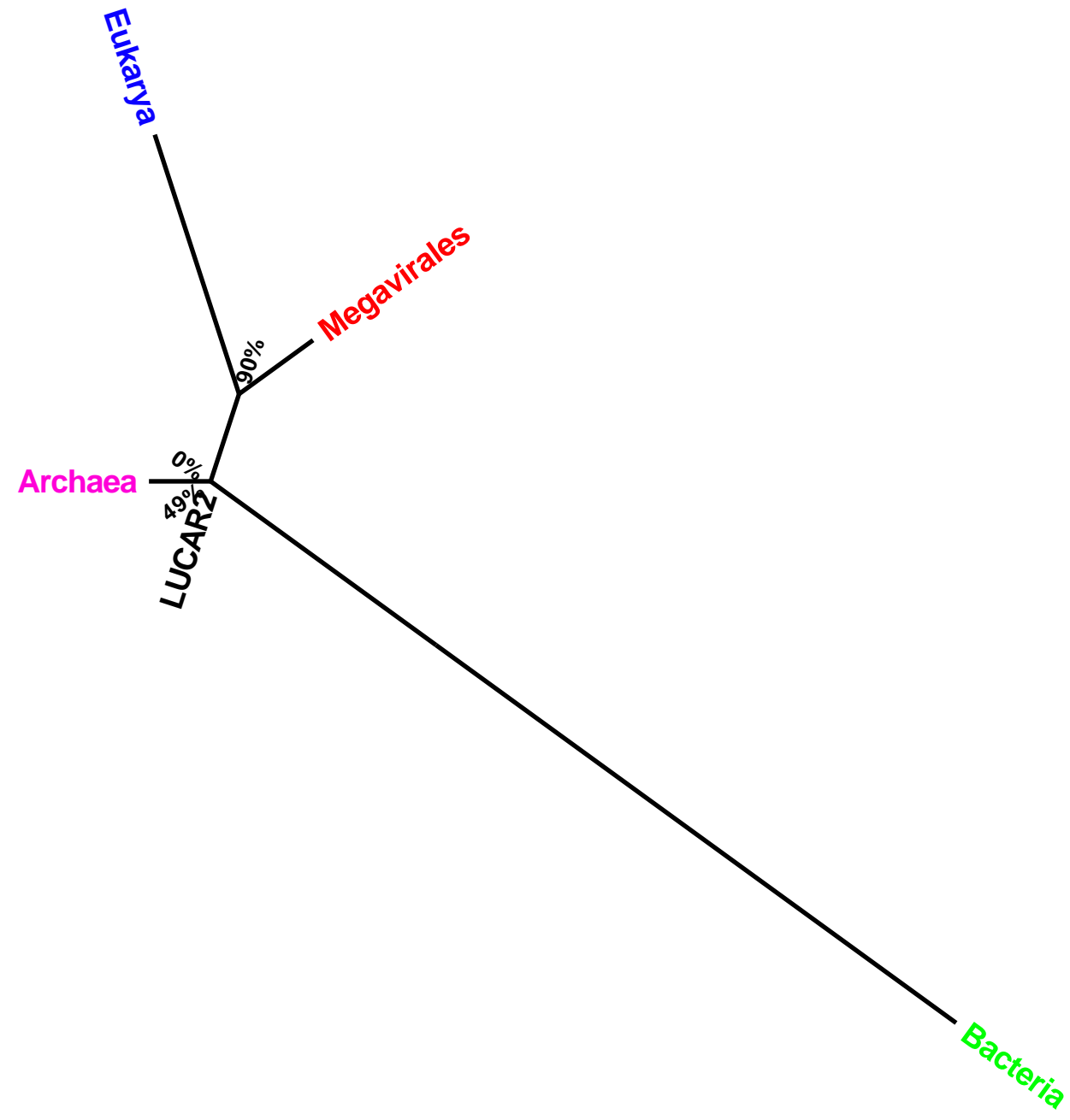

Supplement: Supplementary Data [file supp_evu128_RespRev_Sharma_Raoult_GBE_May2014_Rev_Tree_fig_1_plus_ancestral_3.pdf]

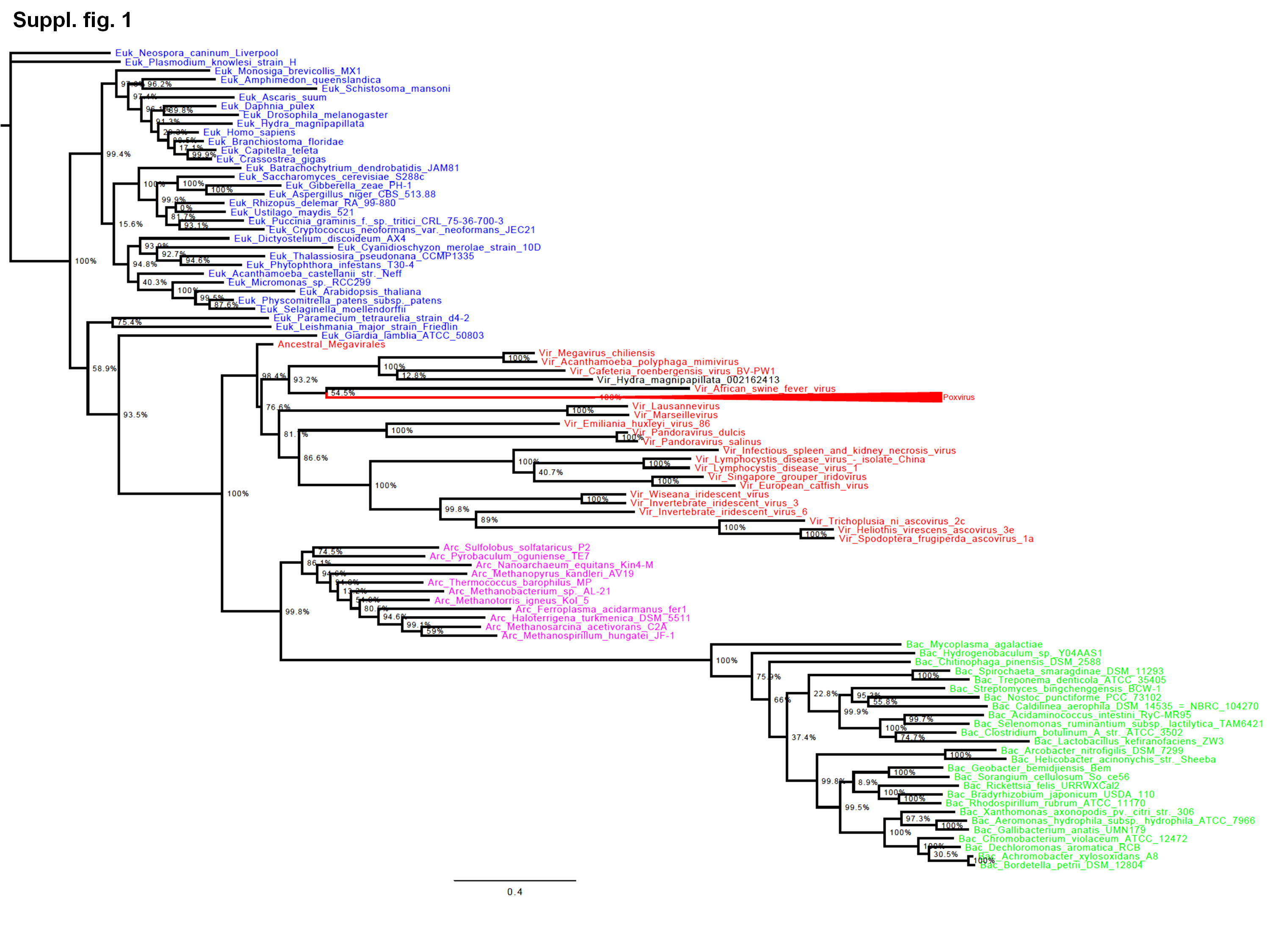

Supplement: Supplementary Data [file supp_evu128_Suppl_fig1_Sharma_Raoult_GBE_May2014_RevvD.tif]

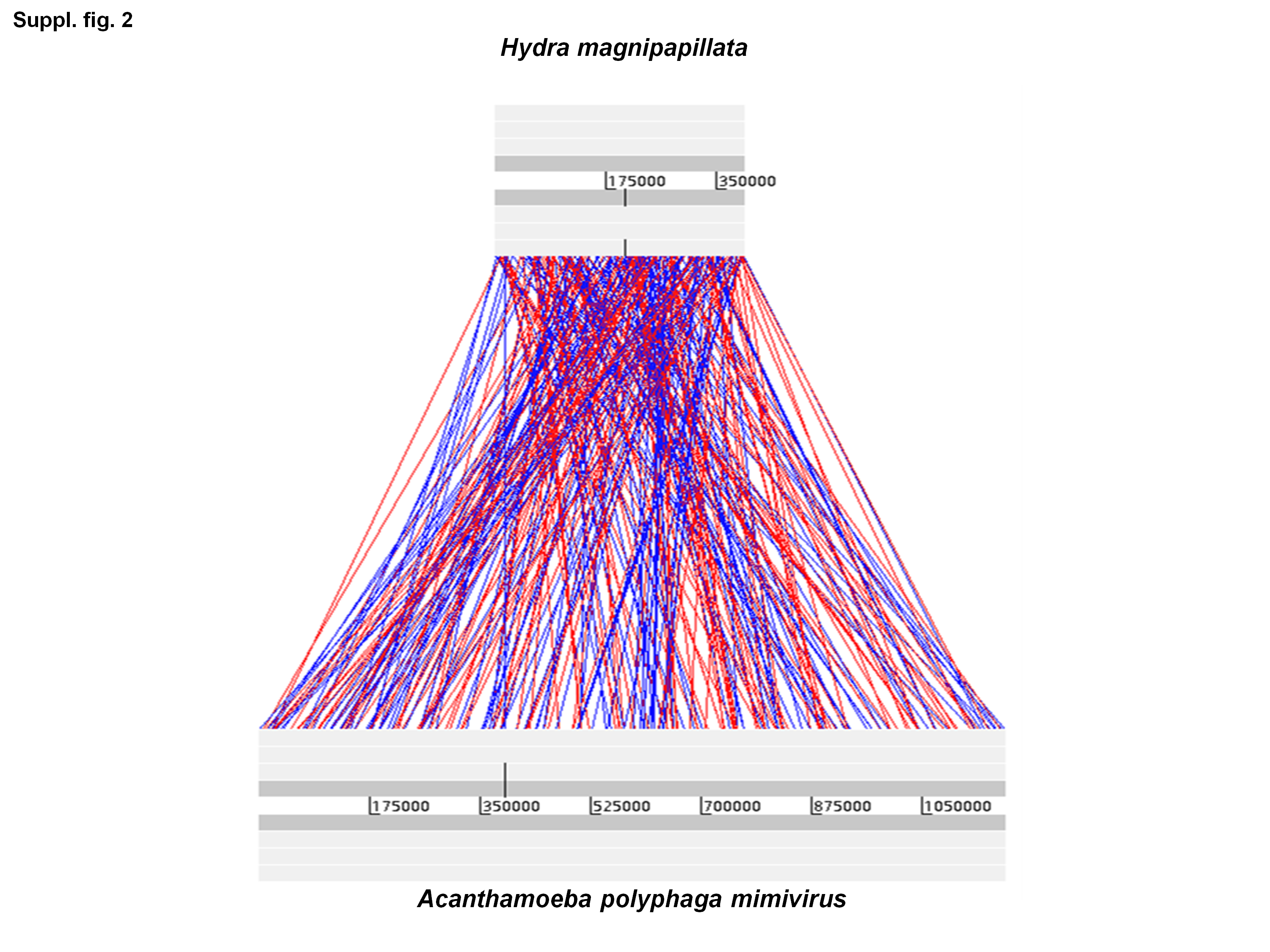

Supplement: Supplementary Data [file supp_evu128_Suppl_fig2_Sharma_Raoult_GBE_May2014_RevvD.tif]

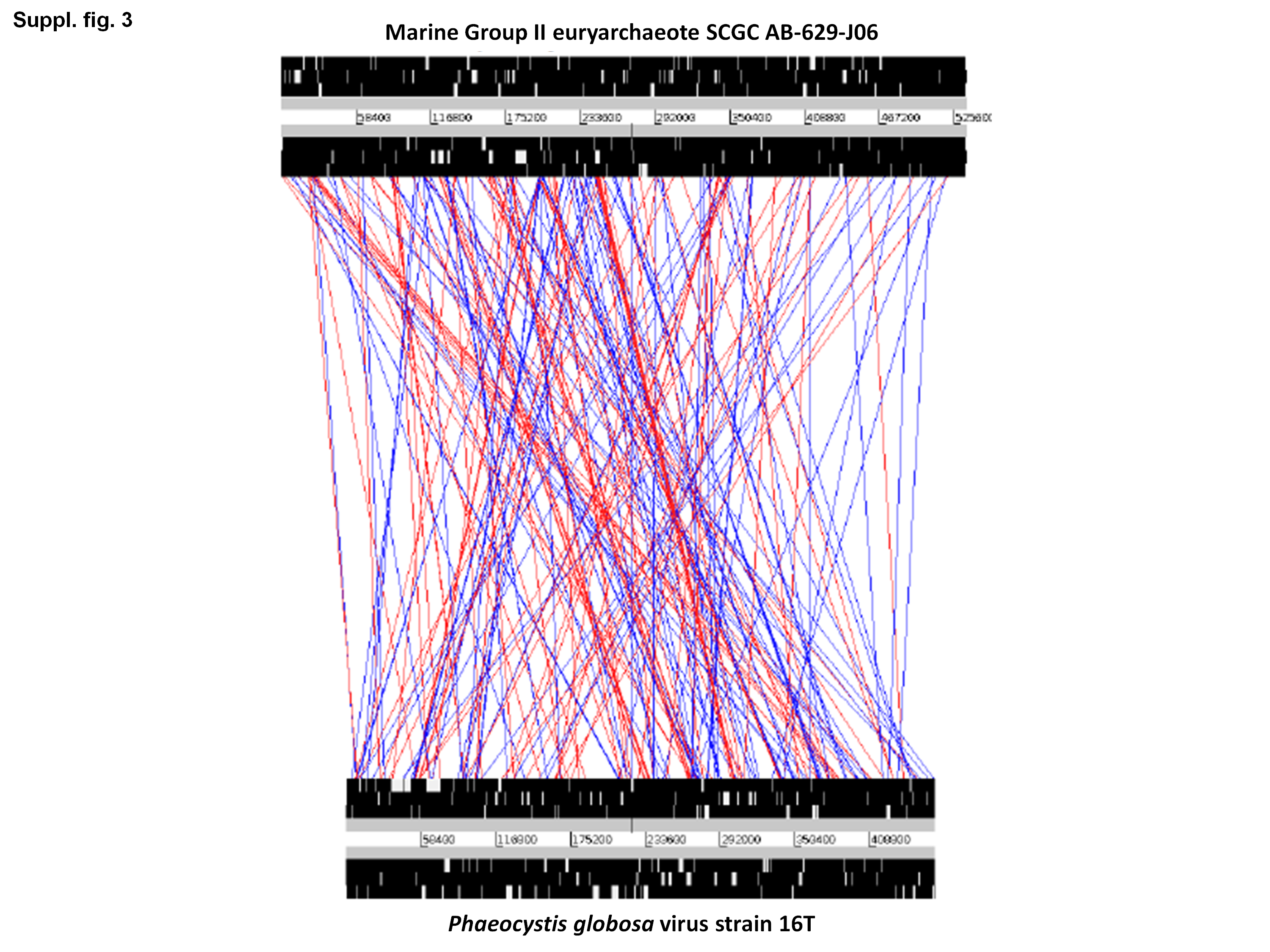

Supplement: Supplementary Data [file supp_evu128_Suppl_fig3_Sharma_Raoult_GBE_May2014_RevvD.tif]

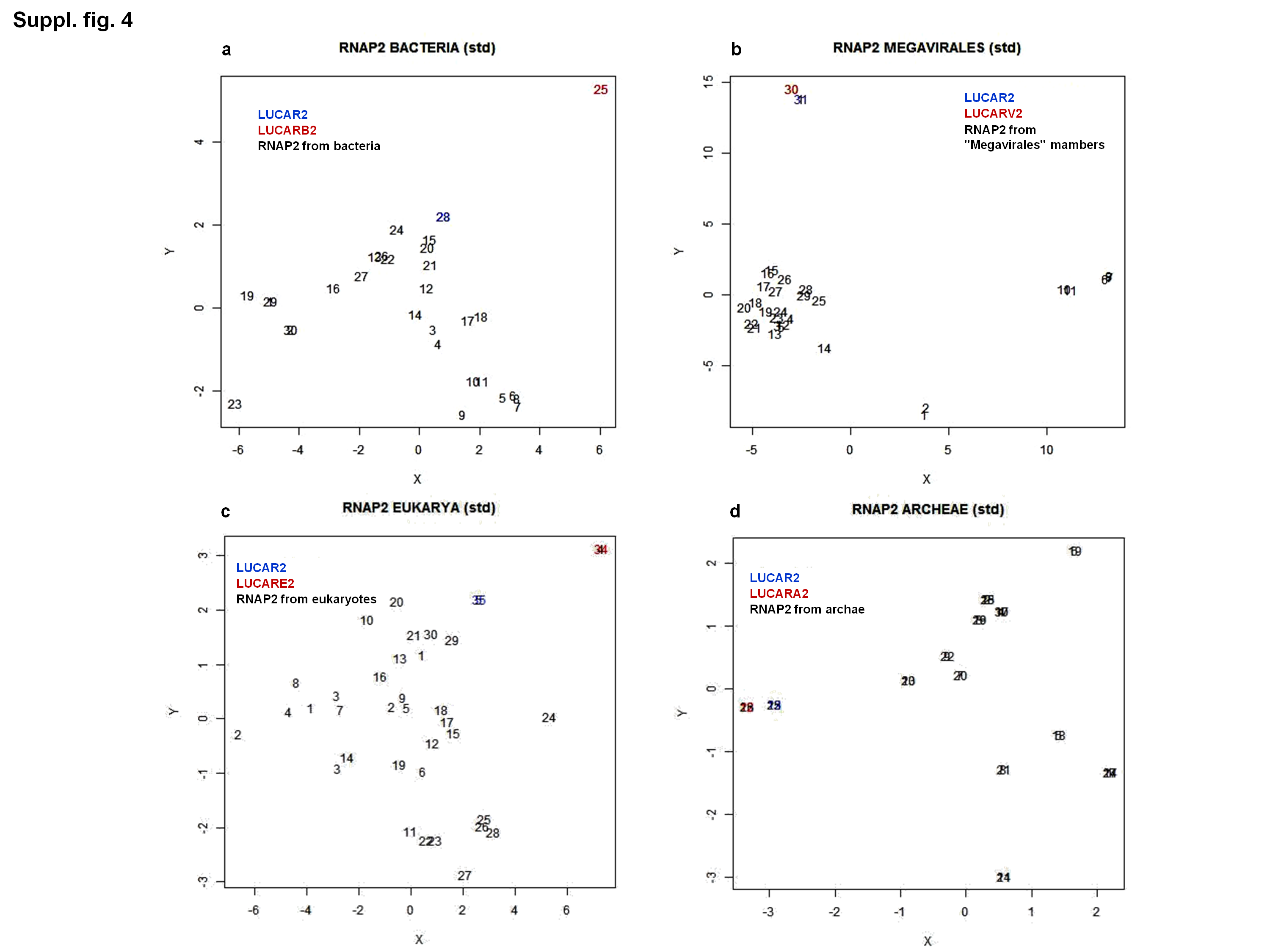

Supplement: Supplementary Data [file supp_evu128_Suppl_fig4_Sharma_Raoult_GBE_May2014_RevvD.tif]

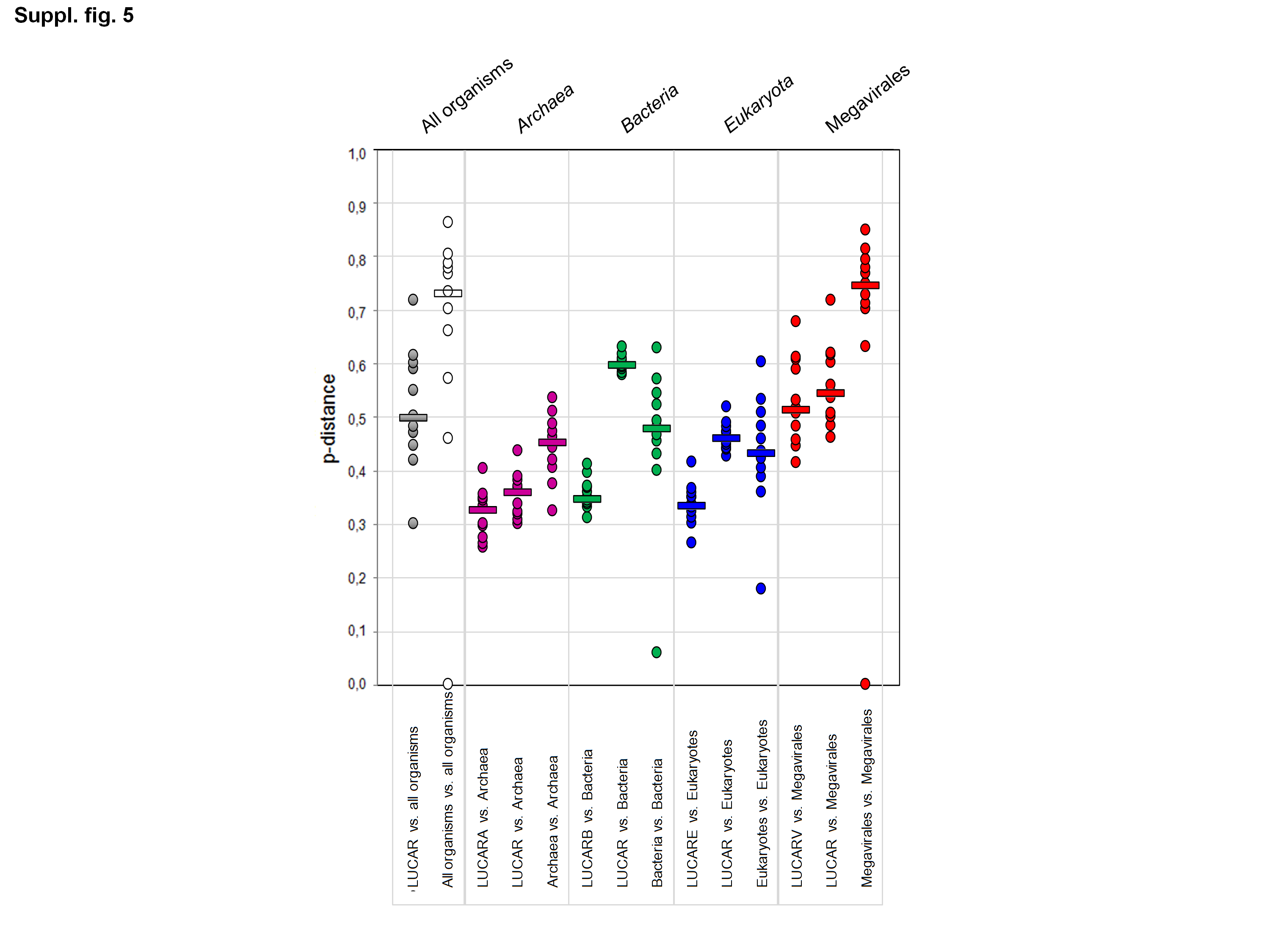

Supplement: Supplementary Data [file supp_evu128_Suppl_fig5_Sharma_Raoult_GBE_May2014_RevvD.tif]

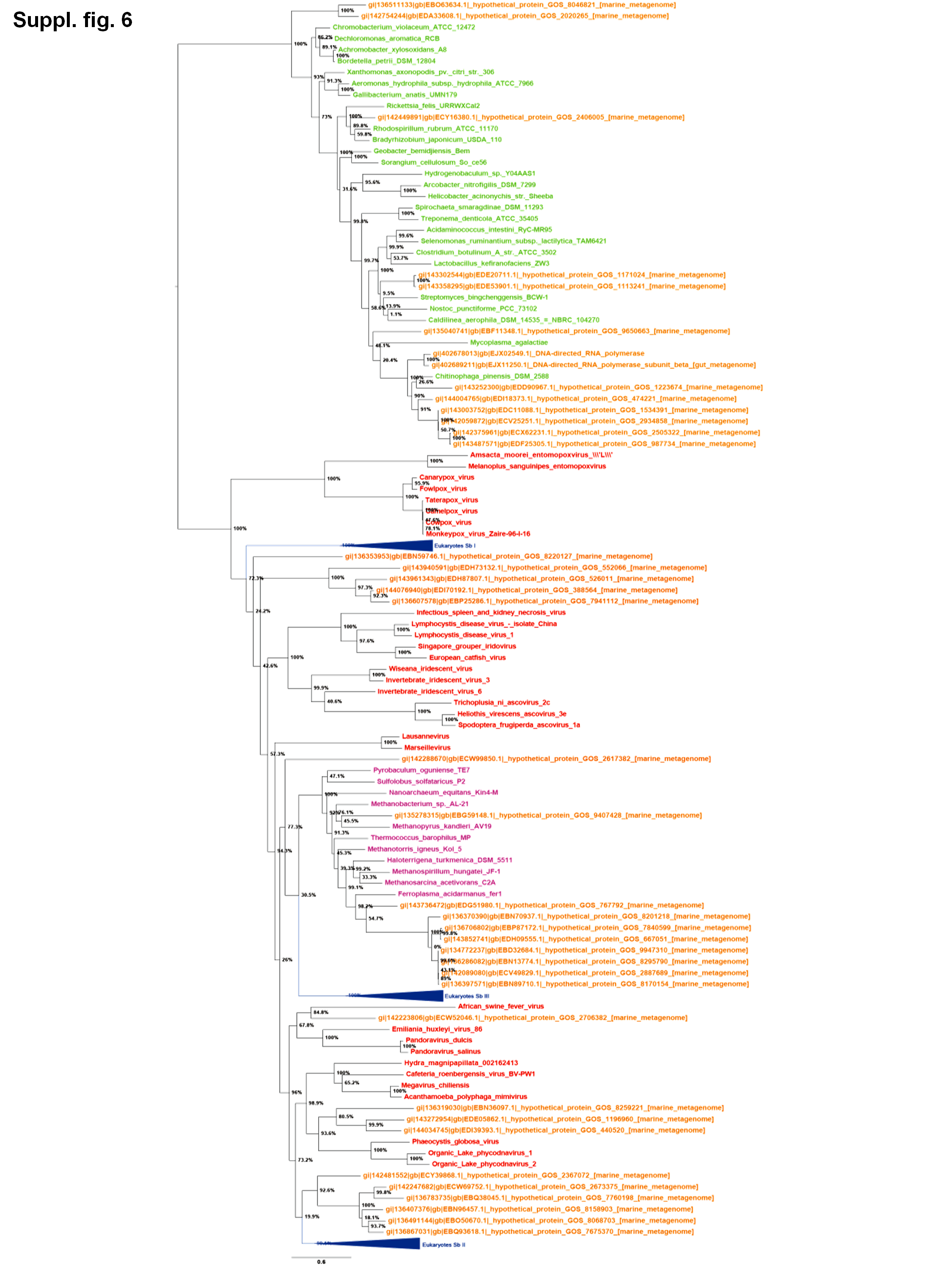

Supplement: Supplementary Data [file supp_evu128_Suppl_fig6_Sharma_Raoult_GBE_May2014_RevvD.tif]

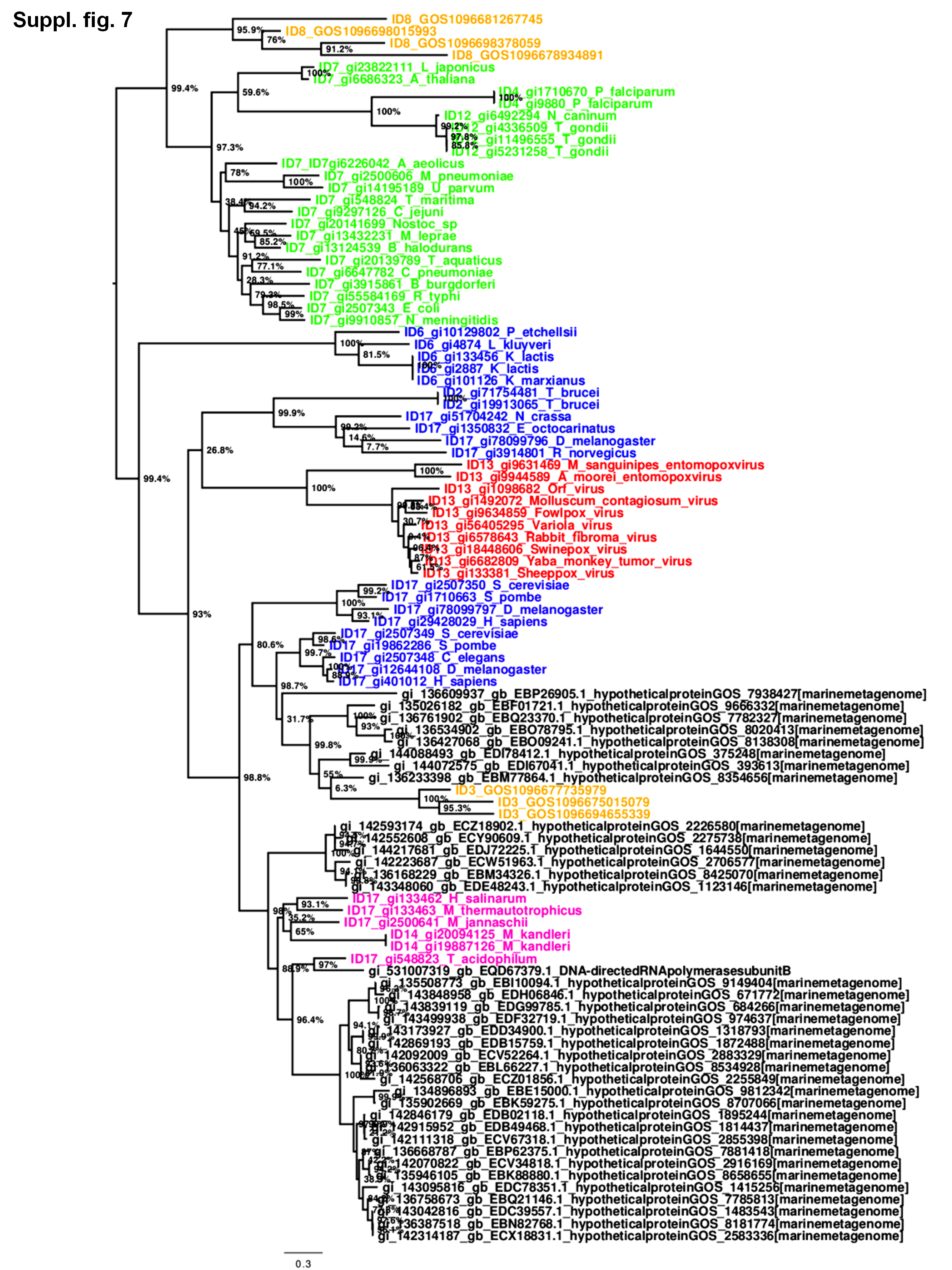

Supplement: Supplementary Data [file supp_evu128_Suppl_fig7_Sharma_Raoult_GBE_May2014_RevvD.tif]
