# Supplementary material for: DNA-Dependent RNA Polymerase Detects Hidden Giant Viruses in Published Databanks
Source: Genome Biol Evol. 2014 Jun 13;6(7):1603–10. doi: 10.1093/gbe/evu128 (PMC4122926; doi:10.1093/gbe/evu128)
Supplement: Supplementary Data [file supp_evu128_Suppl_mat_Raoult_Sharma_GBE_May2014_RevvDl_Unmarked.docx]

**SUPPLEMENTARY MATERIAL**

legends FOR Supplementary figureS

Supplementary figure 1. Phylogeny reconstruction using the maximum likelihood method for DNA-dependent RNA polymerase subunit 2.

The tree corresponds to that shown in Figure 1 except that reconstructed putative ancestral RNAP2 from the “Megavirales” was added (indicated by “ancestral_Megavirales”). Incorporation of reconstructed putative ancestral sequences for the “Megavirales” members did not modify the tree topology, which strengthens the four branch topology. Members of “Megavirales”, *Bacteria*, *Archaea* and *Eukarya* are indicated by red, green, pink and blue fonts, respectively; the extra *Hydra* *magnipapillata* subunit is indicated by a black font. Scale bar represents the number of estimated changes per position.

Supplementary figure 2. BLASTn comparison for one scaffold (gi|262256884|gb|GL020074.1| *Hydra magnipapillata* strain 105 unplaced genomic scaffold HYDRAscaffold_39305) from the *Hydra magnipapillata* draft genome with *Acanthamoeba polyphaga* mimivirus whole genome sequence, using the WebACT tool (Carver et al. 2005).

Supplementary figure 3. BLASTn comparison for the Marine Group II euryarchaeote SCGC AB-629-J06 draft genome (GenBank Accession no. AQVM00000000) against the *Phaeocystis globosa* virus 16T gene repertoire, using the WebACT tool (Carver et al. 2005).

Supplementary **figure 4.** Plots showing multidimensional positioning analyses (MDS) for standardized (Euclidian) p-distances for DNA-dependent RNA polymerase subunit 2 sequences from members of *Archaea*, *Bacteria*, *Eukarya* and “Megavirales”. Each sequence is identified by a number; correspondence between numbers and organism names can be found in Supplementary table 4.

Supplementary **figure 5.** Plots of the distribution of p-distances obtained by pairwise comparisons between reconstructed ancestral sequences for DNA-dependent RNA polymerase (RNAP) subunit 2 from members of *Archaea, Bacteria, Eukarya* and proposed order “Megavirales”and these RNAP sequences, and between these RNAP sequences.

Each circle represents a 10-percentile of p-distances within or across branches of life. Thick lines indicate the median values.

Supplementary **figure 6.** Phylogeny reconstruction using the maximum likelihood method for DNA-dependent RNA polymerase (RNAP) subunit 2 with eukaryotic RNAP2 sequences and best 100 BLAST hits (indicated by a yellow font) for reconstructed putative ancestral RNAP2 in addition to the same datasets than in figure 1. Sequences from members of “Megavirales”, *Bacteria*, *Archaea* and *Eukarya* are indicated by red, green, pink and blue fonts, respectively. Metagenomic sequences are indicated by an orange font. Scale bar represents the number of estimated changes per position.

Supplementary **figure 7.** Phylogeny reconstruction using the maximum likelihood method for DNA-dependent RNA polymerase subunit 2 showing sequences recovered by Wu et al. (Wu et al. 2011) (indicated by a yellow font) from the environmental sequence databases of the Sorcerer II Global Ocean Sampling expedition (GOS) (Yooseph et al. 2007) together with metagenomic sequences fished from the GOS database in the present study.

Sequences from members of “Megavirales”, *Bacteria*, *Archaea* and *Eukarya* are indicated by red, green, pink and blue fonts, respectively. Metagenomic sequences are indicated by a black font. Sequences from Wu et al. are indicated by an orange font. Scale bar represents the number of estimated changes per position.

**LEGENDs FOR SUPPLEMENTARY TABLES**

**Supplementary table 1.** Best BLASTp hits for the *Hydra magnipapillata* strain 105 unplaced genomic scaffold HYDRAscaffold_39305 (GenBank Accession no. GL020074.1) corresponding to proteins from mimiviruses.

**Supplementary table 2.** Best BLASTp hits for the Marine Group II euryarchaeote SCGC AB-629-J06 draft genome (GenBank Accession no. AQVM00000000) corresponding to proteins from mimiviruses.

**Supplementary table 3.** BLASTp hits for contig accession no. KI669605 from *Phytophthora parasitica* strain INRA-310 against proteins from “Megavirales” members.

**Supplementary table 4.** Correspondence between numbers and organism names for multidimensional positioning analyses (MDS) shown in Supplementary figure 4.

**Supplementary table 5.** BLASTp hits using reconstructed ancestral sequence for DNA-dependent RNA polymerase subunit 2 from members of *Archaea*, *Bacteria*, *Eukarya* and proposed order “Megavirales” (LUCAR2) as query against the NCBI GenBank non-redundant protein sequence database (nr).

Reference List

Carver TJ et al**.** 2005. ACT: the Artemis Comparison Tool. Bioinformatics. 21:3422-3423.

Wu D et al**.** 2011. Stalking the fourth domain in metagenomic data: searching for, discovering, and interpreting novel, deep branches in marker gene phylogenetic trees. PLoS One. 6:e18011.

Yooseph S et al**.** 2007. The Sorcerer II Global Ocean Sampling expedition: expanding the universe of protein families. PLoS Biol. 5:e16.
